# Filling the gap: electrochemical access to the missing group IV HfO-based rocksalt compound

**DOI:** 10.1039/d6mh01419d

**Published:** 2026-08-27

**Authors:** Christian Bechteler, Richard I. Todd

**Affiliations:** a Department of Materials, University of Oxford Parks Road Oxford OX1 3PH UK richard.todd@materials.ox.ac.uk

## Abstract

Rocksalt structured group IV transition metal carbides and nitrides (δ-MeX) are well-known materials and are used for various applications. However, the corresponding group IV transition metal monoxides (δ-MeO) are either rarely researched, or, in the case of hafnium, their existence is unknown. In this investigation, electrical loading of yttria-stabilised hafnia was conducted in an inert atmosphere (argon) to achieve electrochemical reduction. Resistive heating of the sample was maintained as the furnace cooled down such that on removing the current, the sample was rapidly quenched, avoiding reoxidation or diffusion-limited phase transformations. XRD, SEM, EDS, and Raman spectroscopy showed that defective rocksalt structure δ-(Y)HfO with a lattice constant of 0.4527 nm was formed and retained at room temperature for the first time. Yttria was rejected from the hafnia-based phase during reduction, leaving a relatively low concentration of yttrium in solid solution. The new compound has a metallic-gold-brown colour and conducts electricity at room temperature (∼0.67 Ω cm). Furthermore, the compound fills the gap in the non-radioactive group IV transition metal carbide, nitride, monoxide rocksalt structure series.

## Abstract 2

Electrochemical reduction and quenching of yttria-stabilised hafnia yield rocksalt structure δ-(Y)HfO at room temperature. Featuring a metallic-gold color and electronic conductivity, it fills the gap in reported group IV interstitial compounds.

## Graphical abstract

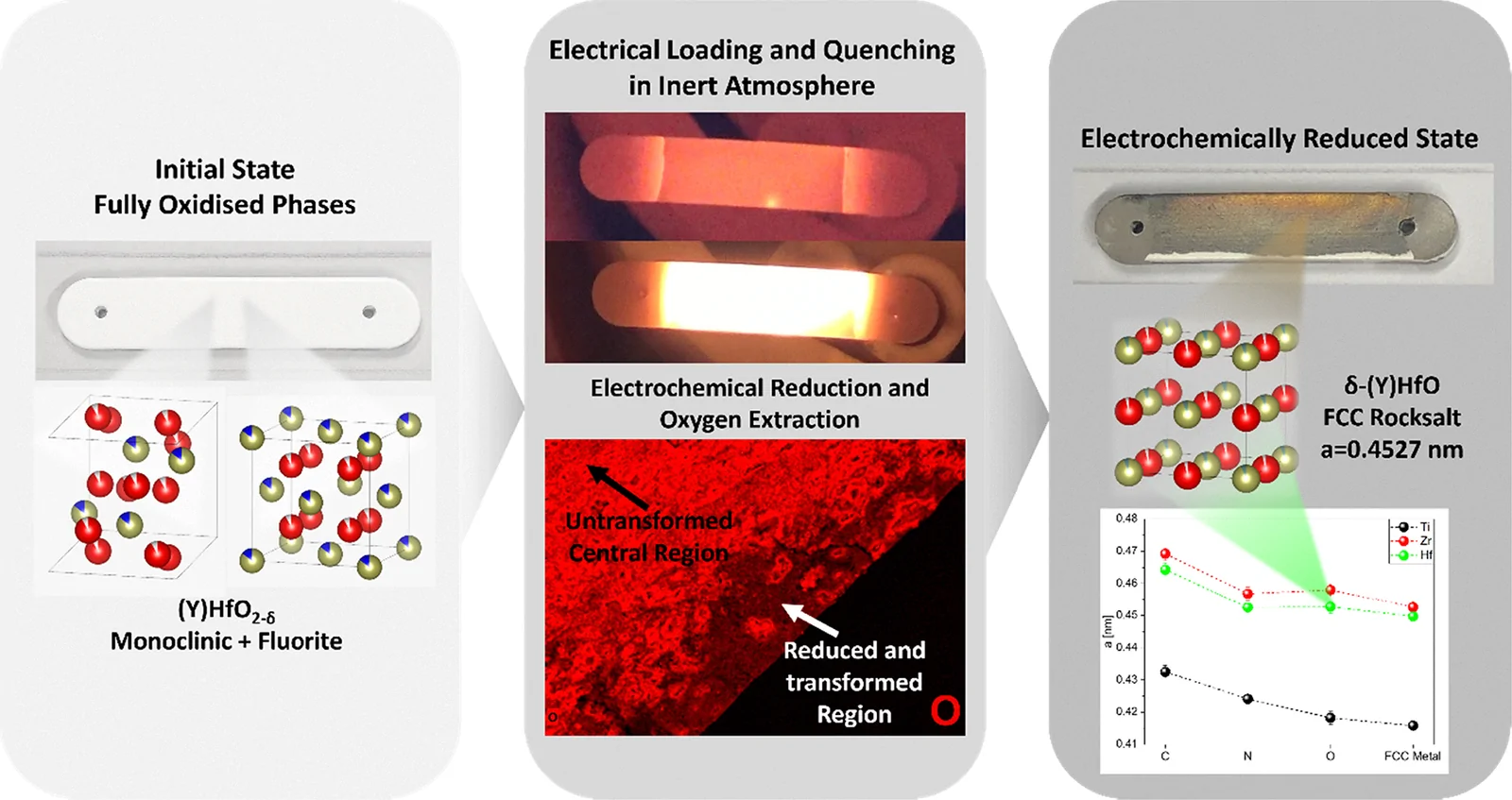

**New concepts**

Rocksalt structured transition metal carbides and nitrides (δ-MeC, δ-MeN) are well-known ceramics, yet the corresponding monoxides – particularly δ-HfO – have remained experimentally elusive. While δ-TiO and impure δ-ZrO have been reported as metastable, defect-rich phases, the absence of δ-HfO represents a “missing link” in the group IV rocksalt series. We bridge this gap through a novel electrical loading technique, utilising aggressive electrochemical reduction to drive oxides into far-from-equilibrium states. By leveraging strong DC fields at elevated temperatures, we manipulate the defect chemistry, successfully synthesising δ-(Y)HfO and retaining it at room temperature. Structurally, δ-(Y)HfO conforms to the interstitial model, with oxygen occupying octahedral sites in an expanded FCC hafnium-based sublattice. Its lattice parameters and metallic conductivity align perfectly with group IV trends, completing the monoxide family. This work establishes field-driven non-equilibrium processing as a transformative strategy for discovering previously inaccessible phases.

## 1. Introduction

Hafnia (HfO_2_), zirconia (ZrO_2_), and titania (TiO_2_) are common group IV transition metal oxides. The yttria-stabilised forms of hafnia and zirconia (Y_*x*_Hf_1−*x*_O_2−0.5*x*_, Y_*x*_Zr_1−*x*_O_2−0.5*x*_) exhibit high ionic but low electronic conductivity, along with useful thermal and mechanical properties, making them attractive for applications such as SOFC/SOEC electrolytes,^1,2^ high-K dielectrics,^3^ and thermal barrier coatings.^4^ Titania is a well-known photocatalyst, semiconductor and biomaterial.

Group IV carbides and nitrides (δ-MeX, Me = Ti, Zr, Hf; X = C, N) crystallise in the rocksalt structure, with lattice parameters set by the radii of Me and X. Thus, δ-MeC have larger unit cells than δ-MeN, and the order for the metallic elements is Zr > Hf > Ti, which is not in order of the periodic table (Ti, Zr, Hf), but can be explained by the extension of the “lanthanide contraction” to Hf, wherein the poor shielding of 4f-electrons allows the outer electrons to be pulled closer to the nucleus, giving it a slightly smaller metallic radius than Zr. These δ-MeX compounds combine high electrical conductivity, hardness (>25 GPa), oxidation and corrosion resistance, and very high melting points (*e.g.* HfC > 3900 °C), classifying them as ultra-high temperature ceramics (UHTCs).^5–7^ δ-MeN phases are particularly conductive and are often superconducting near 10 K, with *T*_c_ tunable by adding a small excess of N or electrons.^7–9^

Rocksalt structured monoxides (δ-TiO, δ-ZrO) were reported as early as the 1950s^10,11^ and continue to be of interest in recent publications.^12–14^ These phases remain poorly understood due to their instability and synthesis challenges, though recent work^13–16^ showed that δ-ZrO can be produced *via* electrochemical reduction of YSZ. YSZ is normally an ionic conductor but at high imposed currents or in an oxygen-free environment, the cathode is unable to incorporate sufficient oxygen to balance the loss of oxygen from the lattice at the anode under an applied DC electric field. The consequent reduction is responsible for the formation of the monoxide. δ-TiO and δ-ZrO exhibit metallic conductivity. The structures are highly defective and do not usually conform precisely to the perfect 1 : 1 stoichiometry. In addition, the recent reports of δ-ZrO are likely impure, containing significant amounts of Y left over from the yttria-stabilised starting material.

The previous reports raise the question of whether a δ-HfO-based substance – the missing group IV monoxide analogue – can also form and be maintained at room temperature. In this study, we investigate the synthesis of a δ-HfO-based rocksalt phase through an electrochemical reduction and quenching process of yttria-stabilised hafnia in inert atmosphere and compare its structure and properties with other δ-MeX compounds.

## 2. Materials and methods

Yttria-stabilised hafnia (YSH) samples were prepared by weighing Y_2_O_3_ (Alfa Aesar, 99.995% pure) and HfO_2_ (Thermo Scientific, 99.9% pure) powders, dispersing them in ethanol with ultrasonication, and homogenising *via* magnetic stirring (10 × 1 min). The slurry was dried in a rotary evaporator, sieved (100 µm), uniaxially pressed (100 MPa), cold-isostatically pressed (200 MPa) into bars (3.5 × 6 × 31 mm^3^), and pre-sintered in air at 1500 °C for 2 h (5 K min^−1^) to 80% relative density, labelled as *Reference*. The theoretical final composition of Y_0.15_Hf_0.85_O_1.925_ was chosen due to its good, anticipated oxygen ion conductivity,^17^ which is required to achieve the electrochemical reduction.

Isothermal electrical loading was performed in an alumina tube furnace at 800 °C, applying a DC field of 150 V (≈75 V cm^−1^) stepwise, as described in ref. 18. After ∼180 s, the current surged, switching to a power-controlled stage at 20 W (∼ 0.03 W mm^−3^). The atmosphere was changed from air to argon (Ar) 120 s later, and the furnace was switched off after a further 100 s, cooling to 50 °C over 2 h while maintaining 20 W Joule heating to the sample. The Joule heating caused the sample temperature to be significantly higher than the furnace temperature at all stages of the process. Power was turned off at 50 °C furnace temperature, quenching the sample rapidly down to the surrounding temperature. Such samples are labelled as *Reduced* in what follows. Quenching is not expected to influence the structure or microstructure of the samples as no displacive phase transformation is predicted or observed. LabVIEW was used to control the power supply (EA-PSI 9750–60; EA Elektro-Automatik GmbH & Co. KG, Germany) and recorded electrical data.

Samples were transferred to an Ar-filled glove box for XRD, conducted with an air-tight rotating holder (2*θ* = 20–120°, 0.04° s^−1^, Cu Kα *λ* = 1.5406 Å, Empyrean, Malvern Panalytical GmbH, Germany). Rietveld refinement in Profex software^19^ considered monoclinic (space group 14, *P*2_1_/*c*), fluorite cubic (space group 225, *Fm*3̄*m*), and cubic yttria Y_2_O_3_ (space group 206, *Ia*3̄) to identify phase concentrations in the *Reference* sample. For simplicity, in apportioning site occupancies, the average composition was used for the fluorite and monoclinic phases. This is not expected to lead to serious errors owing to the small variations in composition according to the phase diagram and the dominance of the Hf in scattering. In addition, rock-salt structured (Y)HfO, with the experimentally observed composition Y_0.07_Hf_0.93_O_0.95_ (space group 225, *Fm*3̄*m*), zincblende HfO (space group 216, *F*4̄3*m*), fluorite cubic HfO_2_ and a 50% reduced fluorite HfO with disordered oxygen vacancies (both space group 225, *Fm*3̄*m*) structures were included to identify the structure and phase concentration of a newly formed phase identified in the *Reduced* material.

Raman spectra were recorded at room temperature using a 50 mW 632.8 nm He-Ne laser (Renishaw System 1000) focused on the sample surface and the resulting fluorescence collected using a × 50 lens.

FEG-SEM (Merlin, Zeiss) and EDS (QUANTAX FlatQUAD, Bruker) characterized the microstructure and elemental distribution; samples were coated with 3 nm Pt.

To accurately determine composition, a manual ZAF correction was applied to the raw EDS k-ratios. The matrix was modeled as a fully dense system, assuming a fluorite-type cubic Y_0.15_Hf_0.85_O_1.925_ structure (*a* = 0.5129 nm) with a theoretical density of *ρ* = 9.6 g cm^−3^ for the *Reference* material and rocksalt HfO structure (*a* = 0.4527) for the reduced phase with a theoretical density of *ρ* = 13.9 g cm^−3^. The atomic number factor (*Z*) was determined by calculating the mean stopping power and backscatter coefficients based on the assumed phase densities. The absorption factor (*A*) was calculated using the mass absorption coefficients (*µ*/*ρ*) for the Hf–M and O–K lines, accounting for the specific X-ray exit path lengths within the respective cubic and rocksalt lattices. The fluorescence factor (*F*) was neglected, as the low excitation energy (3 keV) minimized the probability of secondary characteristic X-ray generation. The correction was performed iteratively until the weight fractions converged, ensuring the calculated chemical composition was self-consistent with the assumed phase densities.

Electrical conductivity of pre-sintered samples before and after electrical loading was measured at room temperature with a digital multimeter, assuming uniform conductivity across the sample cross section.

## 3. Results

### 3.1. Electrical loading

About 180 s after applying the field (max. 75 V cm^−1^, 800 °C furnace temperature), a rapid current rise and thermal emission^20,21^ occurred (Fig. 1a), preceded by hot-spots remote from the electrodes (Fig. 1b and c). Under power control in air, fluctuation in the electrical parameters was small (Fig. 1a) and heating was homogeneous (Fig. 1d) with an estimated average sample temperature of approximately 1050 °C according to the black body radiation model (BBR)^22^ using an emissivity of 0.7.

**Fig. 1:**
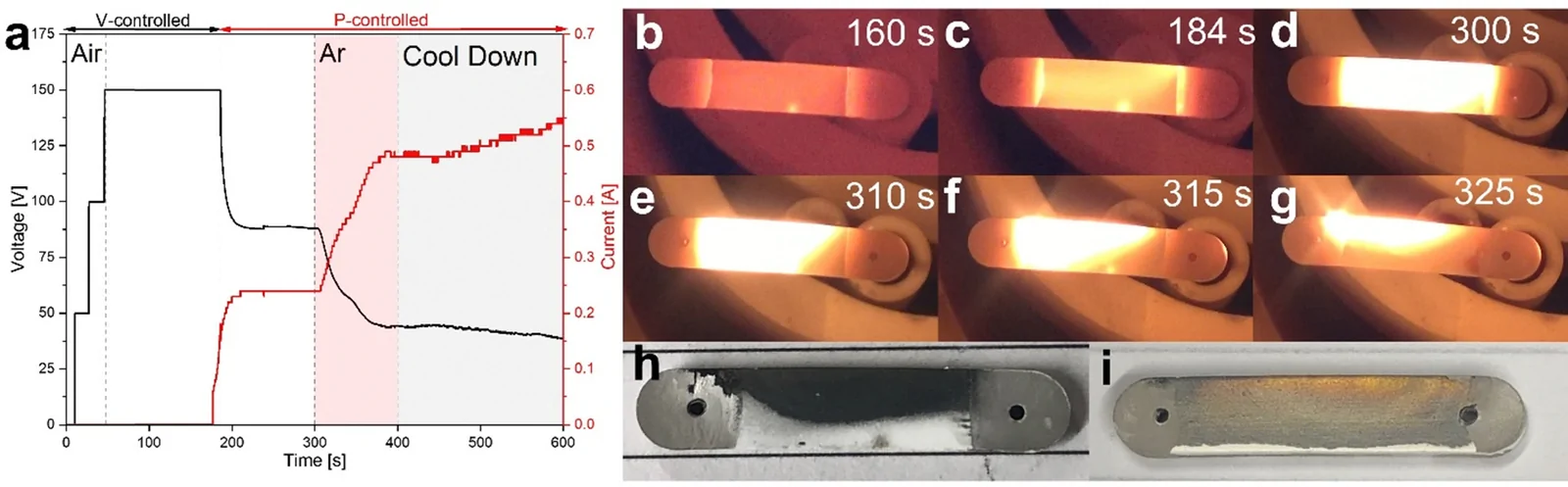
Electrical loading of pre-sintered Y_0.15_Hf_0.85_O_1.925_; (a) voltage (black) and current (red) measured during electrical loading including atmospheric gas (Air or Ar) and start of furnace cool-down; (b–g) sample under electrical load at different stages throughout the process; (h–i) sample after electrical loading and cool-down at room temperature from both sides.

Switching to Ar at 300 s reduced the sample resistance by >75% within 50 s, increased current, and localised heating at the anode (left hand end of the sample in the figures), while hot-spots (Fig. 1g) and electrode-sample-interface heating, particularly on the cathode side (right), disappeared (Fig. 1e–g). Since the surrounding atmospheric temperature is rather low, the plasma formation observed under more extreme circumstances,^18–23^ did not occur here.

After furnace turn-off at 400 s but with power dissipation in the sample still ongoing, the current increased and voltage decreased during cool-down (Fig. 1a), indicating a positive temperature coefficient (PTC) of resistivity.

After approximately 2 h, as the furnace temperature dropped below 50 °C, the electrical power to the specimen was also stopped, which led to a drop in estimated sample temperature from 850 °C to 50 °C within a few seconds. Post-treatment, the sample showed mixed coloration on both sides: white in unaffected regions and black/grey or gold where localised heating had occurred (Fig. 1h–i), including the region where the hot-spot formed before the rapid increase in current (Fig. 1b–c). This is consistent with colour changes previously reported in YSZ.^13^ The greater extent of colouration towards the top of the samples (Fig. 1h–i), is because the current flow tends to concentrate towards the upper side half of the sample^24^ owing to the better contact of the electrode wires at the top side of the holes used to suspend the sample on the wires. The “side” based asymmetry – black on the camera-facing side and gold on the opposite one in this case (Fig. 1h–i),^22^ – does not follow a systematic trend and can vary between different samples treated under similar circumstances. The colour changes from black to gold with ongoing reduction as in YSZ^13,14^ and the observations here are most likely caused by an asymmetric initial current flow.

Electrical loading during furnace cool-down was designed to limit reoxidation and other diffusion-controlled processes, ensuring that the room-temperature state reflects the electrically heated state. This enables identification of structural changes and electrochemically induced property modifications.

### 3.2. SEM and EDS

SEM images of the near-surface microstructure of the *Reduced* sample, shown in Fig. 2(a) and (b), reveal the formation of particles 50–100 nm in dimensions within both large and small grains, unlike the centre region of the sample (Fig. 2c), or the *Reference* sample (Fig. S1), which do not show such particles.

**Fig. 2:**
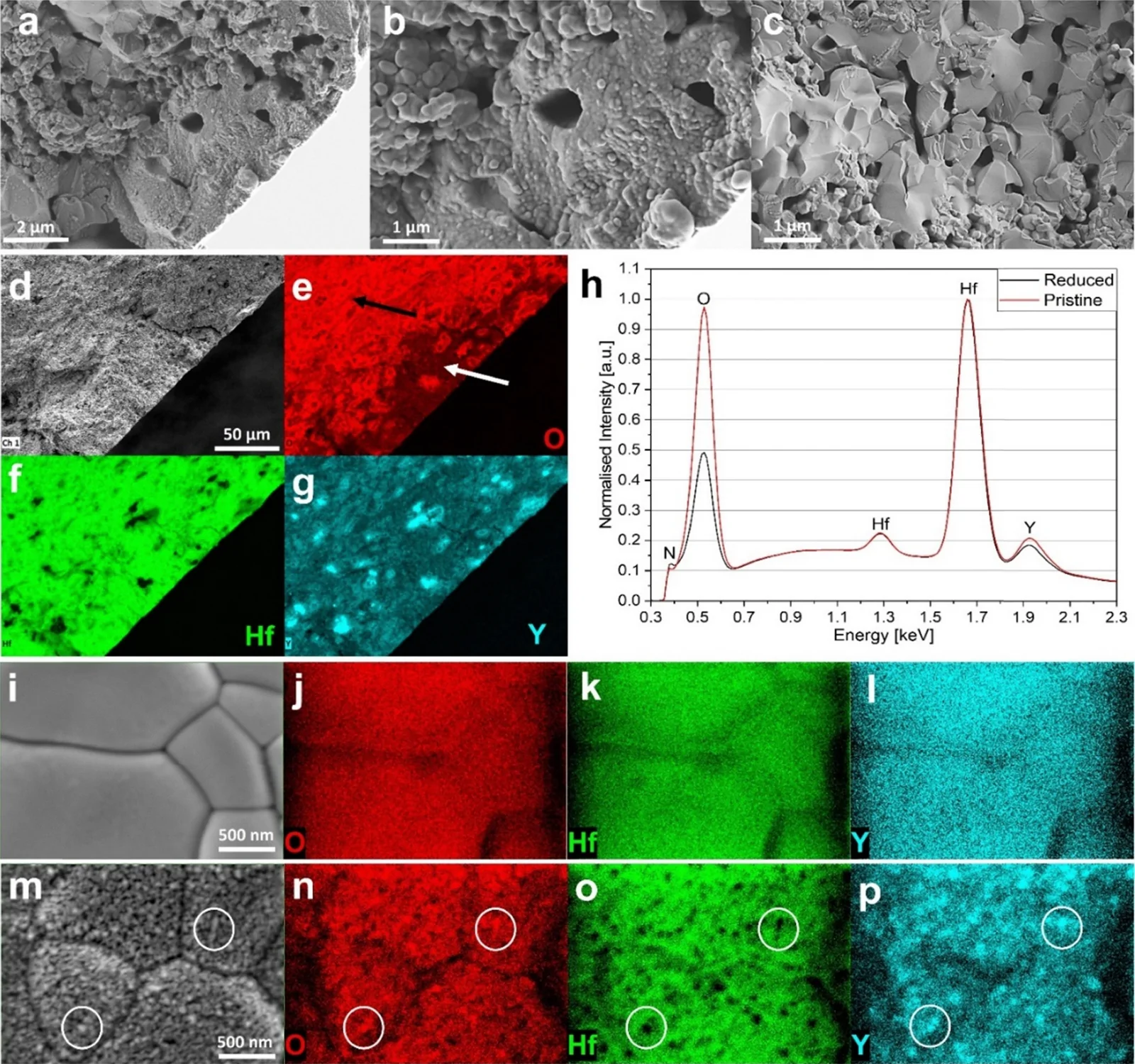
SEM images of the fractured cross section of reduced yttria stabilised hafnia (a) and (b) close to the surface, and (c) closer to the less reduced centre of the cross section; (d)–(g) show a SEM image and the elemental distribution maps of a fracture surface of reduced yttria stabilised hafnia, close to the original sample surface and with oxygen (O) in red, hafnium (Hf) in green, and yttrium (Y) in blue; (h) shows two point spectra taken from reduced yttria stabilised hafnia close to the reduced oxygen-depleted surface region highlighted in (e) by a white arrow, and a less reduced oxygen-rich region highlighted by a black arrow; (i)–(l) show a SEM image and the elemental distribution maps of the surface of *Reference* yttria stabilised hafnia sample and (m)–(p) show the surface of reduced yttria stabilised hafnia.

Elemental distribution mapping (Fig. 2d–g) reveals a pronounced oxygen-depleted zone beneath the surface of the *Reduced* sample. This O-depletion is corroborated by EDS spectra normalised to the Hf signal (Fig. 2h), which contrast the distinct chemical profiles of the near-surface and core regions. However, the depth of this oxygen-depleted zone is spatially non-uniform; as evidenced in Fig. 2e. The mean depth of the zone is 10–20 µm but the maximum depth in the micrographs is 70 µm, whilst some localised oxygen-rich domains persist at certain surface locations and within the oxygen depleted layer.

ZAF-corrected EDS was performed on the core of the *Reduced* sample. The resulting composition of Y_0.14_Hf_0.86_O_1.94_ shows excellent agreement with the mean nominal composition of Y_0.15_Hf_0.85_O_1.925_. The atomic compositions are within 0.5% of the nominal value in each case, which is consistent with the statistical errors calculated, giving confidence in the EDS analysis.

This newly formed phase in the surface region exhibited a ZAF-corrected composition of Y_0.07_Hf_0.93_O_0.95_. This reveals a significant rejection of yttrium from the transforming lattice – halving the local Y concentration compared to the initial fluorite Y_0.14_Hf_0.86_O_1.94_ phase. The rejected yttrium is concentrated in the small, Y-rich particles evident in this region (Fig. 2g), which are identified as yttria in section 4.3. The Kanaya–Okayama range indicates a resolution of ∼50 nm for EDS of the new phase, which is comfortably sufficient for the measurements to reflect only the surface layer in which it is found, and to avoid excessive contamination of the signal from the Y-rich particles. Furthermore, the ZAF corrected composition suggests the formation of oxygen vacancies in the rocksalt δ-(Y)HfO phase since the ratio of oxygen/cations is 0.95.

The SEM images and EDS maps from *Reference* (Fig. 2i–l) and *Reduced* (Fig. 2m–p) sample surfaces are consistent with the near-surface cross sections. The *Reference* sample shows well-defined grain boundaries between smooth and homogeneous larger grains, whereas the *Reduced* sample exhibits a rougher appearance consisting of fine 50–100 nm grains or particles over the whole surface. Some are again Y- and O-rich, showing the rejected Y_2_O_3_ (circled in Fig. 2m–p), while most of the surface is Hf-rich and O-depleted, with significantly less Y, showing the newly formed rocksalt δ-(Y)HfO phase.

### 3.3. XRD-characterisation and Rietveld refinement

Background-corrected XRD patterns of the *Reference* and *Reduced* samples are shown in Fig. 3a), with predicted patterns for the refined structures in Fig. 3b) and the Hf-based model structures shown in Fig. 3(e)–(g).

**Fig. 3:**
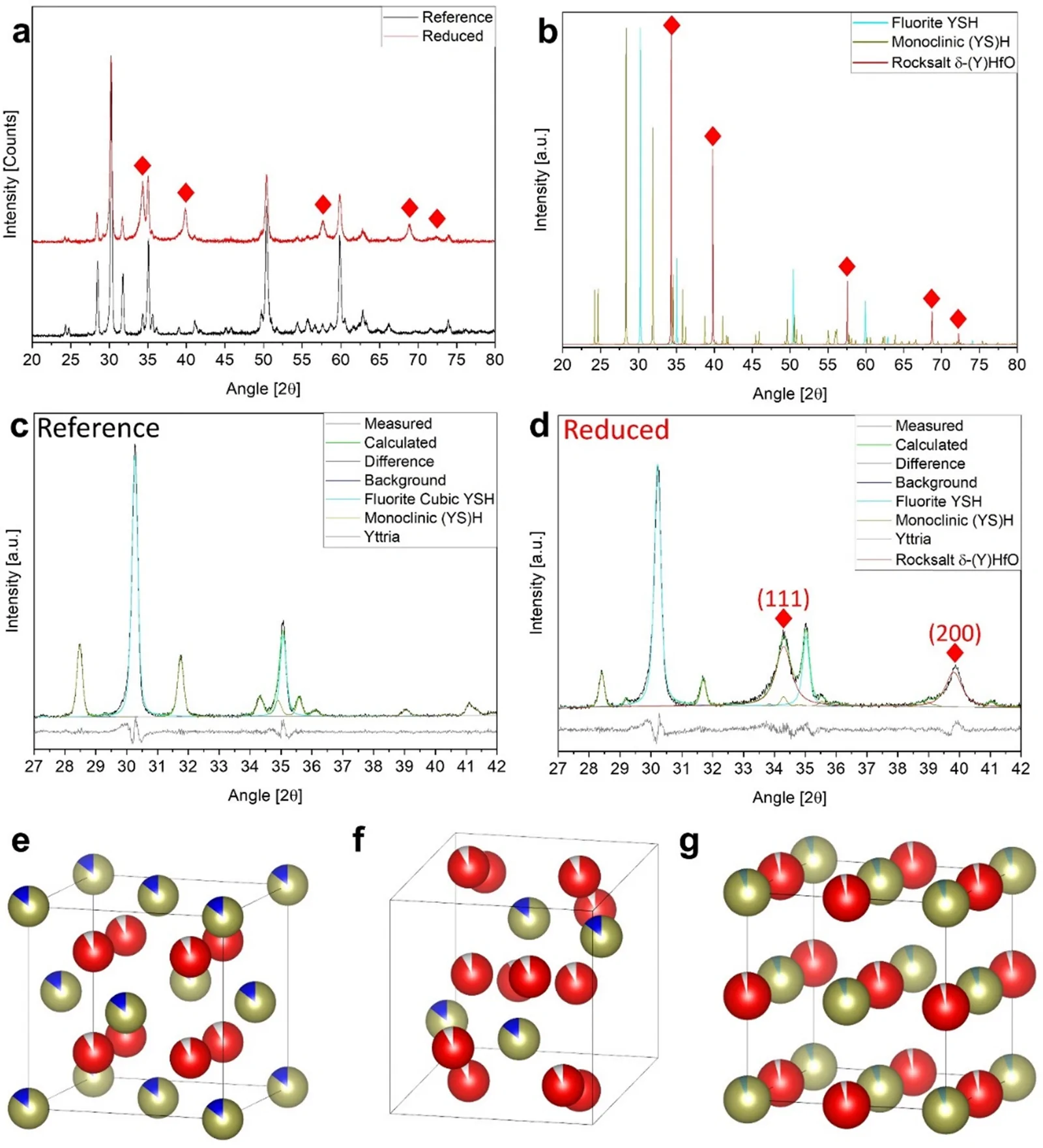
(a) XRD pattern of pre-sintered yttria stabilised hafnia before (*Reference*) and after electrical loading (*Reduced*). In the reduced sample rocksalt phase peaks appear, highlighted with red diamond symbols; (b) predicted peak locations and intensities for fluorite YSH, monoclinic (YS)H, and rocksalt δ-(Y)HfO; Rietveld refined XRD pattern of (c) *Reference* and (d) *Reduced* yttria stabilised hafnia; (e) fluorite YSH (Y_0.14_Hf_0.86_O_1.93_); (f) monoclinic (yttria stabilised) hafnia; (g) rocksalt δ-(Y)HfO (Y_0.07_Hf_0.93_O_0.95_) unit cells used for Rietveld refinement, O in red, Hf in gold, Y in blue and white represents vacancies.

Rietveld refinement of the *Reference* sample XRD pattern identified a tri-phasic mixture consisting of monoclinic (Y)HfO_2_, cubic fluorite YSH, and undissolved yttria (Y_2_O_3_) from the original powder mixture (Fig. 3c and Table 1). The measured lattice parameter of the fluorite YSH phase (*a* = 0.5129 nm) is consistent with previously reported values^25,26^ for the nominal composition used here. Overall, the refined model showed a good fit (*χ*^2^ = 1.12), supporting the validity of these results.

**Table 1:** Rietveld refined lattice parameters and phase composition of pre-sintered *Reference* and electrochemically *Reduced* yttria stabilised hafnia, including phase composition detected by ZAF-corrected EDS and refined XRD

Phase	*Reference*		*Reduced*		Composition (EDS/XRD)
	Lattice parameter [nm]	Concentration [wt%]	Lattice parameter [nm]	Concentration [wt%]	
Fluorite YSH	*a* = 0.5129	78.3	*a* = 0.5125	65.0	Y_0.14_Hf_0.86_O_1.94_
Monoclinic	*a* = 0.5121 *b* = 0.5153 *c* = 0.5306 *β* = 99.166°	21.0	*a* = 0.5120 *b* = 0.5149 *c* = 0.5309 *β* = 99.175°	10.9	(Y_*X*_)Hf(_1−*X*_)O_2_
Yttria	*a* = 1.0591	0.7	*a* = 1.0592	2.1	Y_2_O_3_
Rocksalt δ-(Y)HfO	—	—	*a* = 0.4527	22.0	Y_0.07_Hf_0.93_O_0.95_

The observed presence of the monoclinic phase in the *Reference* material also agrees with the expectation from the Y_2_O_3_–HfO_2_-phase diagram,^27–30^ which suggests that around 20 mol% of Y_2_O_3_ would be needed for full fluorite stabilisation at room temperature,^30^ more than twice the amount of Y_2_O_3_ used here (8.1 mol%). According to the phase diagram, the composition used in this investigation is fully fluorite at the maximum sintering temperature of 1500 °C but partly transforms to monoclinic during cool-down to room temperature, as observed.

The *Reduced* sample exhibited a simultaneous ∼10 wt% decrease in the monoclinic (Y)HfO_2_ and fluorite YSH phases, and an increase from 0.7 wt% to 2.1 wt% of Y_2_O_3_ (see also Fig. S2, SI) within the XRD-analysed volume – characterised by a calculated attenuation length of 7.7 µm (calculation see SI) – summarised in Table 1. Notably, this transition occurred with negligible deviation in the lattice parameters of the primary monoclinic, fluorite cubic YSH, and yttria phases. The Y_2_O_3_ can be associated with the Y-rich particles in Fig. 2g and p. However, the emergence of distinct Bragg reflections in the *Reduced* diffraction pattern (Fig. 3a), signify the formation of a new phase.

Rietveld refinement using the EDS composition of the reduced zone near the surface (Fig. 3d) indicates that these additional peaks in the *Reduced* material correspond to rocksalt δ-Y_0.07_Hf_0.93_O_0.95_ with a lattice parameter of 0.4527 nm, accounting for a ∼22 wt% fraction, mainly formed from the monoclinic and fluorite cubic YSH phases, both showing a decreasing ratio. The structural model is further validated by a robust fit (*χ*^2^ = 1.16) as is also demonstrated by the intensities of the main peaks in Fig. 3(d). Sensitivity analysis demonstrates that the fit quality degrades significantly if a perfectly stoichiometric rocksalt δ-HfO structure, or another type of FCC structure, such as fluorite structured hafnia, half-reduced fluorite, or zincblende, is considered (Table T1, SI). The comparison of the lattice parameters of this phase with trends in related group IV rocksalt structured compounds in section 5.3 give further confidence in its identification.

### 3.4. Raman spectroscopy

Raman spectra from different regions of the *Reduced* sample, shown in Fig. 4, reveal distinct structural changes. The white region of the sample, similar to the *Reference* material (Fig. S3), shows strong monoclinic hafnia peaks at 149 and 501 cm^−1^ alongside various other peaks that can mostly also be associated with monoclinic hafnia.^31,32^ These disappear in the black/grey region (Fig. 4a).

**Fig. 4:**
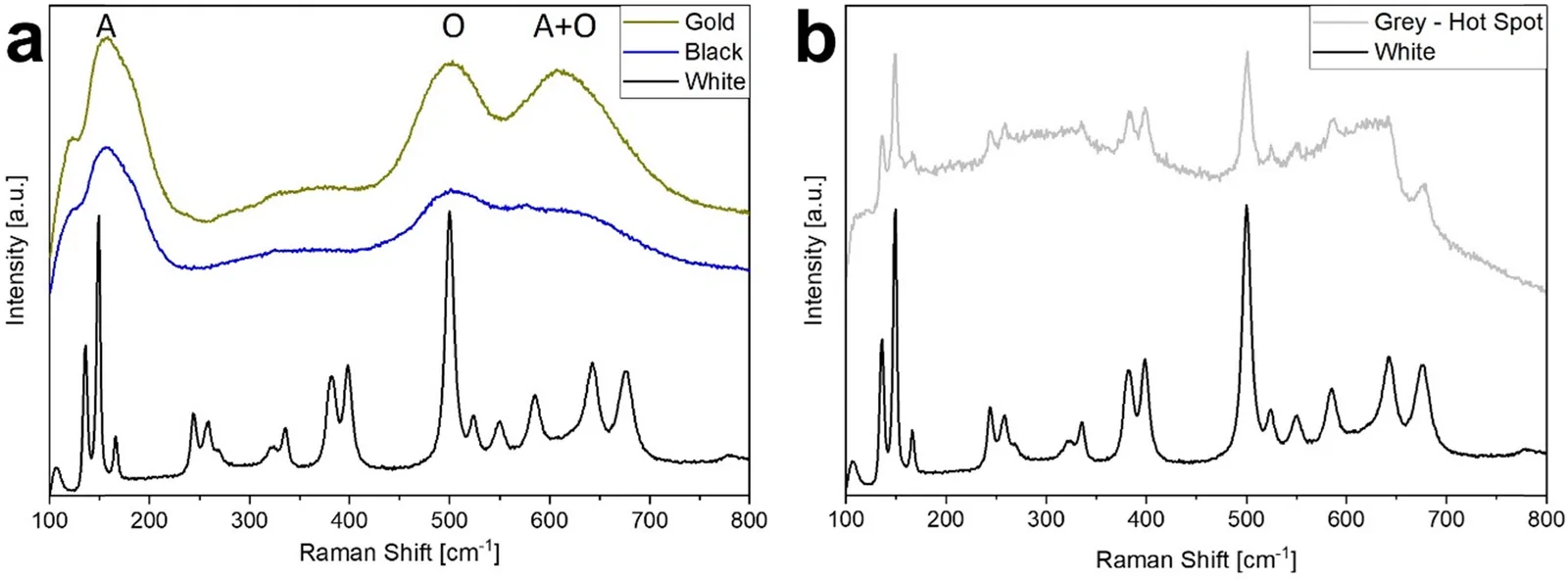
Raman spectra of (a) electrically loaded and electrochemically reduced Y_0.15_Hf_0.85_O_1.925_ in the white, black, and gold region of the sample; (b) hot-spot and the surrounding white regions of the sample.

In the gold region, broad peaks at 158, 501, and 609 cm^−1^ appear, consistent with a defective rocksalt δ-(Y)HfO structure, as ideal rocksalt would be first order Raman-inactive.^33^ Off-stoichiometric rocksalt δ-HfN shows a comparable spectrum with an acoustic peak around 150 cm^−1^, an optical peak around 500 cm^−1^ and an optical + acoustic mode at 650 cm^−1^.^34,35^

Near the hot-spot region (Fig. 1b), monoclinic peaks persisted but were weaker compared with the rather pronounced background than in the white regions (Fig. 4b). This suggests that the hot-spot is more disordered and possibly that some of the monoclinic (Y)HfO_2_ has been converted to other phases.

### 3.5. Electrical resistivity

For the unreduced *Reference* sample, the room-temperature resistance exceeded the range of the multimeter used. After electrochemical reduction, the resistance of the *Reduced* sample dropped to 9.5 Ω between the electrode holes, corresponding to a nominal resistivity of ∼0.67 Ω cm, the same order of magnitude as reduced YSZ containing FCC δ-ZrO.^13^

## 4. Discussion

### 4.1. Local hot spot formation

A local hot spot was observed on the sample surface, not directly linked to the electrodes, even at low current levels before the current increased rapidly. At low currents, conduction is mainly ionic, but electrochemical reduction generates vacancies and other defect structures which gradually shift it towards electronic conduction.^13–36^ For YSZ, a closely related ion conductor, Kirchheim has suggested that in air, electronic conduction dominates at the ends of the specimen, whilst the central portion remains ionically conducting, giving the possibility of cycling oxygen through the atmosphere *via* the sample surfaces.^37^ The hot spot may represent a localised site where the incorporation or emission of atmospheric oxygen at the surface was concentrated.^34^ The reduced character of the surface material at this point, as shown by the blackening, and its closer proximity to the cathode, suggest that the spot is cathodic in character.

This interpretation is also supported by the fact that the hot spot disappeared shortly after the atmosphere was changed from air to Ar (Fig. 1g) thus preventing the incorporation of atmospheric oxygen at the surface. Under these conditions, electronic conduction becomes dominant over the whole length of the specimen. Similar considerations may explain the reduction in heating at the interface between the conductive paste at the cathode and the sample on removing the oxygen in the atmosphere.

### 4.2. The rocksalt δ-(Y)HfO phase

#### 4.2.1. Phase identification and evidence

The formation of the δ-(Y)HfO phase following O-extraction by electrochemical reduction is robustly supported by a synergy of compositional, structural, and microstructural evidence. Collectively, these results provide strong evidence for this phase identification, as follows:

#### 4.2.2. Crystallographic integrity

Rietveld refinement yielded an excellent fit for the δ-(Y)HfO phase (space group *Fm*3̄*m*) with a lattice parameter of *a* = 0.4527 nm. Alternative FCC Bravais lattice structures provided significantly poorer fits to the experimental data as shown in Table T1 in the SI.

#### 4.2.3. Stoichiometric consistency

ZAF-corrected EDS analysis (Fig. 4h) of the Hf-rich domains reveals a corrected composition of Y_0.07_Hf_0.93_O_0.95_, close to the 1 : 1 ratio of metal to non-metal elements in the rocksalt structure.

#### 4.2.4. Lattice scaling

As detailed in section 5.3 below, the lattice parameter determined is entirely consistent with established trends for group IV transition metal δ-MeX compounds (X = C, N, O).

Further confidence in our conclusions is given by noting that combining the mean cation ratio of the 22 wt% of the new phase with composition of Y_0.07_Hf_0.93_O_0.95_ and the extra 1.4 wt% Y_2_O_3_ compared with the *Reference* sample (Table 1) is Y_0.15_:Hf_0.85_, which is identical to the nominal composition in terms of the cations. Our conclusions from EDS and XRD taken together, thus correctly account for the cation content in the *Reduced* sample.

Taken together, this convergence of independent characterization techniques strongly supports the idea that the electrochemical process has produced FCC rocksalt δ-HfO-based material with a lattice parameter of 0.4527 nm for the first time.

#### 4.2.5. Phase stabilization and microstructure

While the existence of the δ-phase is clear, its specific nature is influenced by the electrochemical environment and the parent stoichiometry. Rietveld analysis quantifies that δ-(Y)HfO constitutes ∼22 wt% of the analysed volume. The remaining 78 wt% originates from the better-known monoclinic and fluorite hafnia, and yttria phases. Considering the cross-sectional O map in Fig. 2e and the X-ray attenuation length of 7.7 µm, it is unlikely that the X-rays diffracted from these conventional phases came only from the O-rich regions of the reduced surface zones. Instead, the majority of these diffracted X-rays probably come from the whiter, unreduced regions of the surface shown in the macroscopic views in Fig. 1h–i, which were also included in the area analysed.

#### 4.2.6. Raman activity, defect chemistry and phase stability

While an ideal rocksalt structure is first-order Raman-inactive due to its centrosymmetry,^38^ our spectra exhibit broad vibrational features, which are remarkably similar to those of related δ-MX materials (where M is a transition metal and X is O, N, or C). These observed modes are activated by symmetry-breaking lattice defects,^35–39^ specifically the oxygen vacancies (V_O_) and the substitution of some Hf by Y, as shown by the EDS results.

Furthermore, the observed chemical composition of Y_0.07_Hf_0.93_O_0.95_ coupled with the appearance of Y_2_O_3_ particles shows that the transition from the initial fluorite structure to the electrochemically reduced δ-phase is accompanied by a strong expulsion of Y^3+^ ions from the lattice, indicating that the solubility limit of Y in δ-HfO is 7% on the metallic sublattice.

Similar dopant expulsion has also been observed in the electrochemical reduction of yttria-stabilized zirconia (YSZ) to form an analogous rocksalt phase. Further research is needed to determine whether the presence of the Y or O-vacancies is necessary for the structural stability of these phases.

#### 4.2.7. Electrical conductivity transition

This phase transformation is accompanied by a dramatic shift in electrical behaviour. The sharp decrease in room temperature resistivity to 0.67 Ω cm reflects a transition from the predominantly ionic conduction of pristine YSH to the electronic conduction characteristic of the δ-MeO state, as is also known for δ-ZrO.^13–16^ This transition aligns with the general behaviour of the group IV monoxides, nitrides, and carbides (δ-MeO, δ-MeN, and δ-MeC)^7–41^ and their metallic bonding character.

These findings are of particular relevance for applications operating under extreme reductive loading, such as memristors, high-k dielectrics, and solid oxide electrolysis cells (SOECs).

### 5. Filling the gap

The lattice parameters of known δ-MeX compounds can be described in terms of an “interstitial compound” structural model which features a complex blend of metallic, covalent, and ionic bonding. The lattice parameter of the δ-(Y)HfO-phase (*a* = 0.4527 nm) reported here for the first time is consistent with the corresponding trends. According to this model, the δ-HfX structure is based on the FCC metal (Me) with the smaller, non-metal (X) atoms occupying the octahedral interstices of the structure. Although all three Me's (Hf, Zr, Ti) are hexagonal as elements at room temperature, their FCC lattice parameters can be estimated from their metallic radii *r* as 4 × *r*/√2.

The results for the theoretically calculated metallic FCC lattice parameters (FCC Metal) are shown in Fig. 5 and Table 2 alongside all known δ-MeX (Me = Ti, Zr, Hf; X = C, N, O) lattice parameters, including the result for δ-HfO-based phase from the present work. It is evident that only small expansions of the metallic lattice are needed to accommodate the non-metal atoms, and the extent of the expansion generally ranks in order of the size of the non-metal ions (C > N > O), although the nitrides and oxides are very similar for Zr and Hf, probably because the size of the interstice is comparable to the size of the X atoms so that not much expansion is needed. The lattice parameters of the δ-MeX compounds closely follow the trends in the metallic FCC lattice parameter in every case, further supporting the identification of δ-HfO-based rocksalt phase in this work. Coupled with the room temperature electrical conductivity common to all the other reported δ-MeX, it is clear that the δ-HfO-based phase reported here fills the gap in the non-radioactive group IV transition metal interstitials previously existing for this class of compounds.

**Fig. 5:**
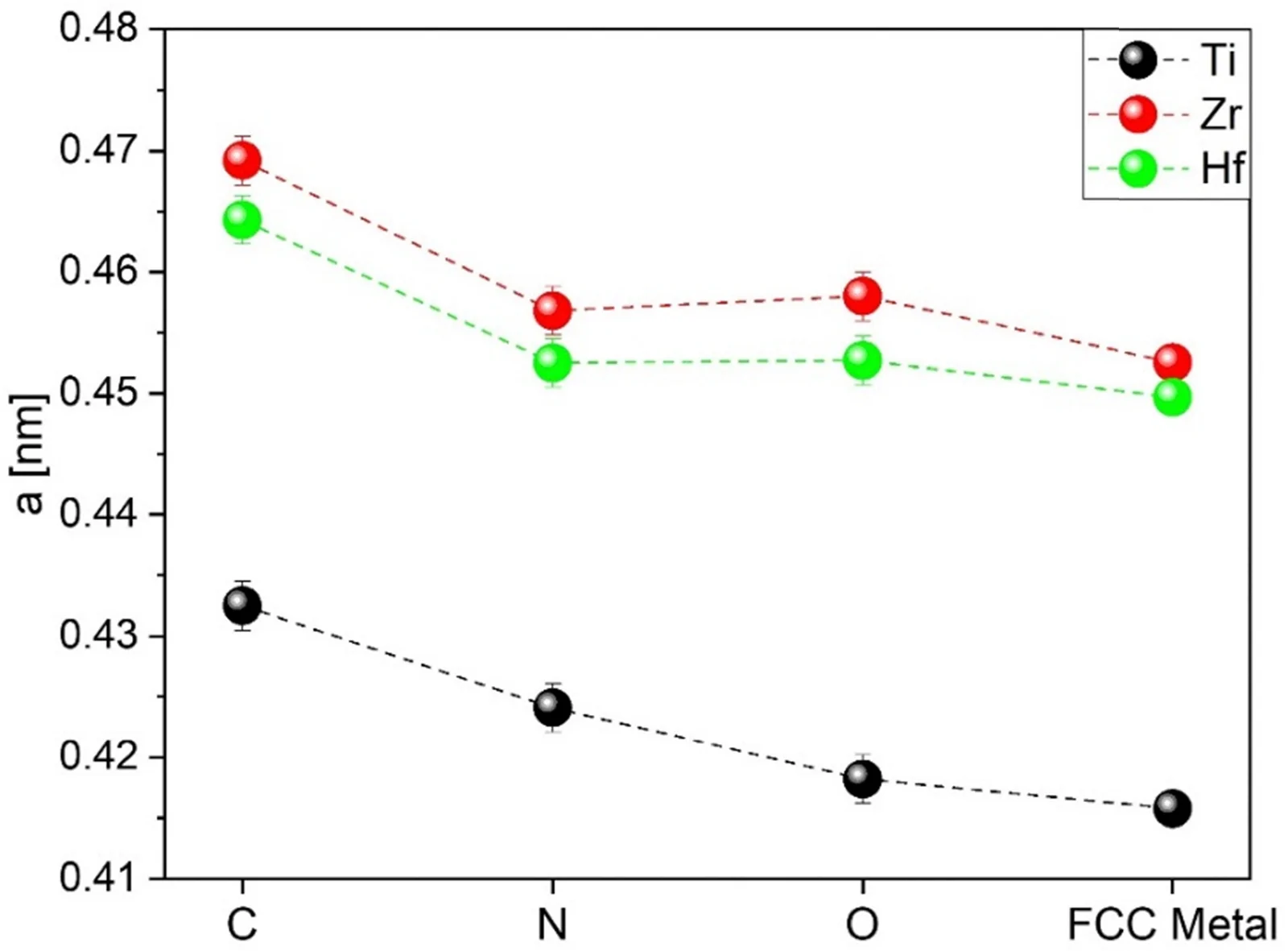
Lattice parameters of all known δ-MeX (Me = Ti, Zr, Hf; X = C, N, O) based structures as well as the theoretically calculated FCC Metal lattice parameters for pure metals based on their metallic radii.

**Table 2:** FCC rocksalt group IV transition metal based intermetallic δ-MeX compounds (Me = Ti, Zr, Hf; X = C, N, O) including their lattice parameters in nm and references. For the theoretically calculated FCC metal phases, the references for the metallic radii are given

	Ti	Zr	Hf
C	0.4325^40^	0.4692^42^	0.4643^5^
N	0.4241^43^	0.4568^44^	0.4525^45^
O	0.4182^10^	0.4580^13,14^	0.4527 [This work]
FCC Metal	0.4158^46^	0.4526^46^	0.4497^47^

## 6. Conclusions

1. The crystal structures, phase contents and microstructures in yttria-stabilised hafnia have been investigated before and after DC electrical loading of pre-sintered samples followed by quenching to room temperature, all in argon (Ar). This novel transformative strategy allows the field-driven synthesis of non-equilibrium phases and materials, which are otherwise inaccessible.

2. The resulting electrochemical reduction of yttria-stabilised fluorite and monoclinic hafnia leads to the formation of rocksalt structured δ-(Y)HfO with a lattice parameter of 0.4527 nm and chemical composition of Y_0.07_Hf_0.93_O_0.95_. The defects in the structure (Y atoms in solid solution and O vacancies) cause it to be Raman-active.

3. The electrical properties, crystal structure, and lattice parameter are all consistent with the trends in previously reported group IV transition metal δ-MeX carbides, nitrides and monoxides. The δ-HfO-based phase synthesised here for the first time therefore represents the last missing interstitial compound in this series.

## Conflicts of interest

There are no conflicts to declare.

## Supplementary Material

MH-013-D6MH01419D-s001

## Data Availability

Data supporting the findings of this study are available within the main manuscript and the supplementary information (SI). Supplementary information is available. See DOI: https://doi.org/10.1039/d6mh01419d.
