# Supplementary material for: Filling the gap: electrochemical access to the missing group IV HfO-based rocksalt compound
Source: Mater Horiz. 2026 Aug 27;13(20):10305–14. doi: 10.1039/d6mh01419d (PMC13679731; doi:10.1039/d6mh01419d)
Supplement: Supplementary file 1 [file MH-013-D6MH01419D-s001.pdf]

Supplementary Information (SI) for Materials Horizons.
This journal is © The Royal Society of Chemistry 2026

Supplementary Information

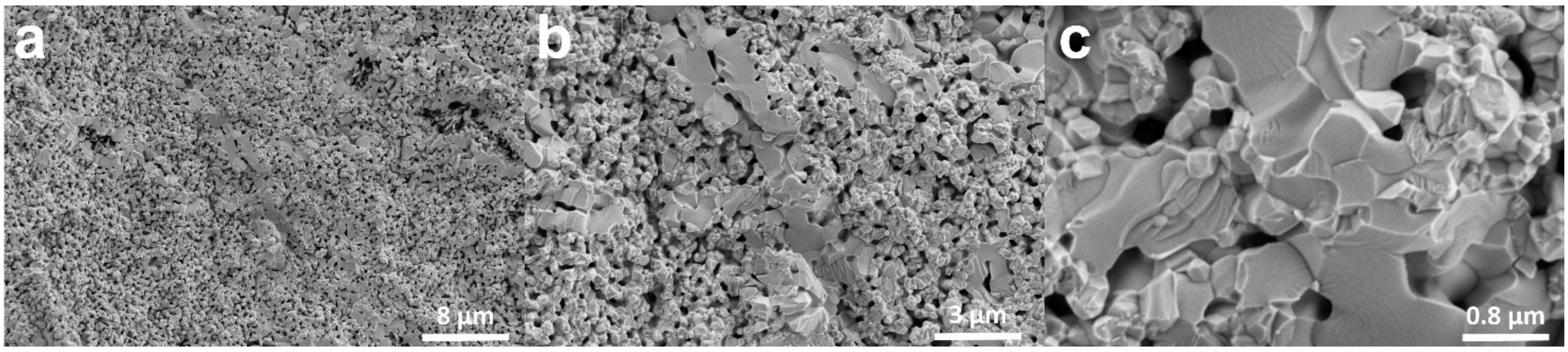

Figure S1: SEM images of a fractured near-centre cross section of a Reference pre-sintered $Y_{0.15}Hf_{0.85}O_{1.925}$ sample without electrical loading at various magnifications.

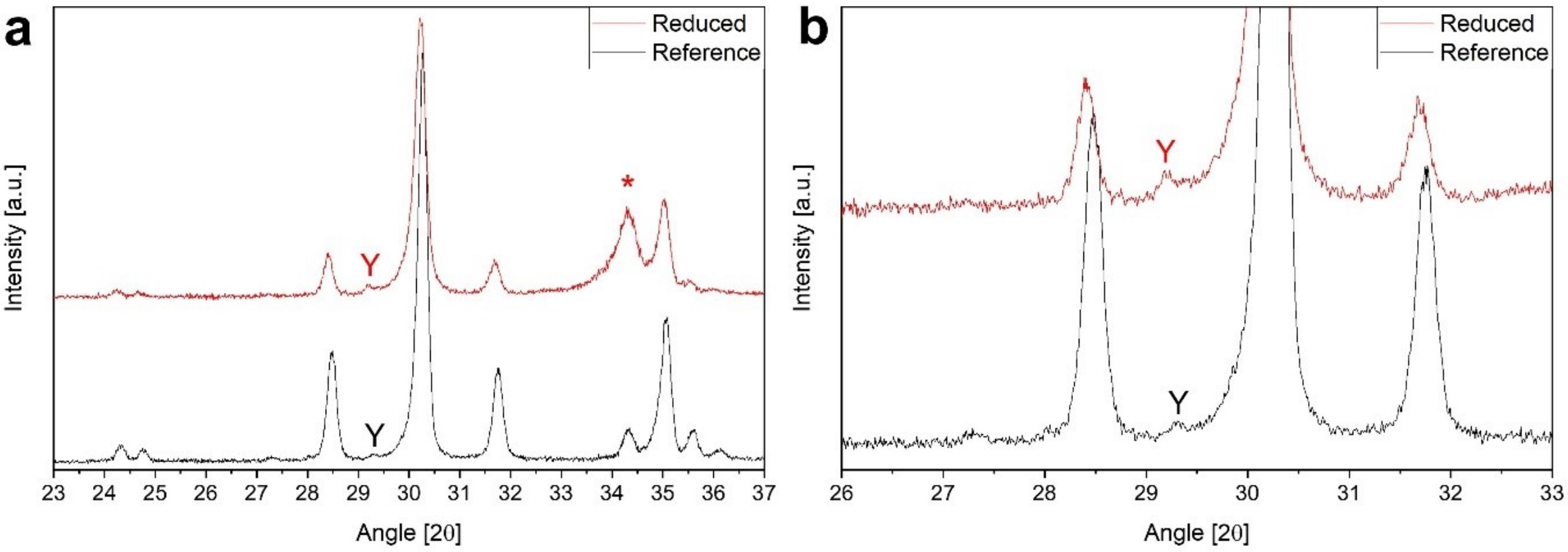

Figure S2: XRD patterns of pre-sintered (Reference) and electrochemically reduced (Reduced) yttria-stabilised hafnia showing the new δ-HfO phase (*) as well as the most intense peak for unreacted yttria (Y2O3) labelled with Y.

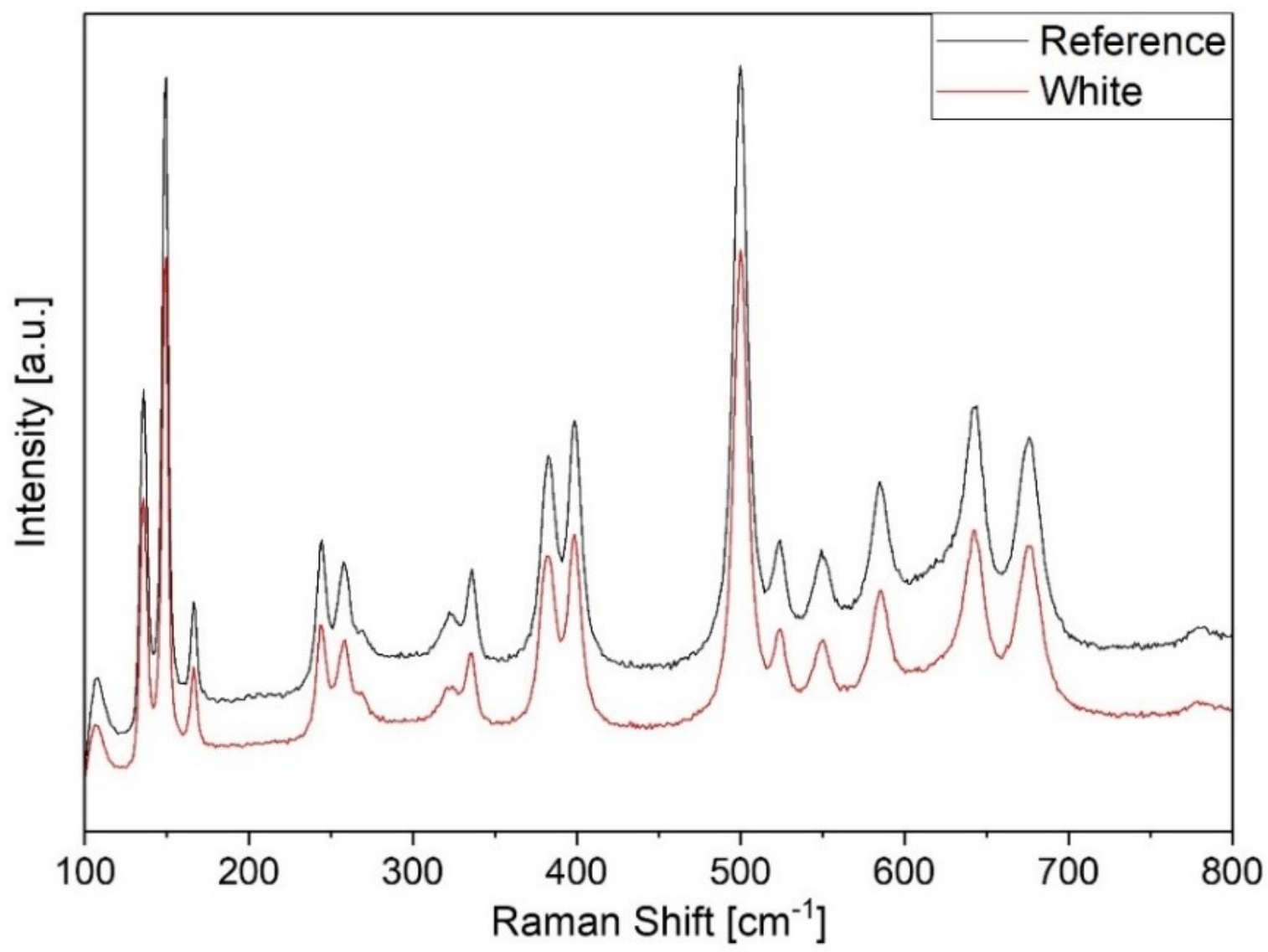

Figure S3: Raman spectra from the Reference sample (black) and the White section of the Reduced sample (red).

Calculation of penetration depth / attenuation length of XRD radiation

Mass-attenuation at ~ 8.05 keV energy of CuKα, taken from NIST data base:

Hf: $\mu/\rho \approx 157.1$ cm²/g

O: $\mu/\rho \approx 11.63$ cm²/g

Mass fractions in HfO_2 210.49 g/mol:

Hf = 178.49 / 210.49 ≈ 0.8480

O = 32.00 / 210.49 ≈ 0.1520

Compound mass attenuation coefficient based on previous results:

$\mu/\rho = 0.8480\times157.1 + 0.1520\times11.63 \approx 135.0$ cm^2/g

Density of HfO_2 (if 100 % dense) $\rho \approx 9.68$ g/cm³

Linear attenuation coefficient:

$\mu = (\mu/\rho)\times\rho \approx 135\times9.68 \approx 1.31\times10^3$ cm^{-1}

Attenuation length:

$1/\mu \approx 7.7$ µm

Table T1: Comparison of various FCC structures, including XRD peak intensities of {111}, {200}, and {220} peaks, and the goodness of fit $\chi 2$ of the refined model including the various FCC structures.

**Structure**	**Intensity [%]**			χ^2
	**{111} Peak**	**{200} Peak**	**{220} Peak**	
HfO_2 Fluorite 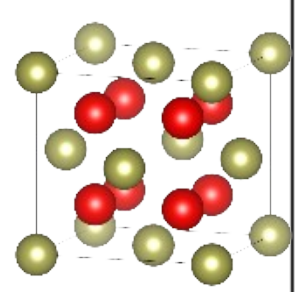	100	29.3	28.7	2.62
HfO Fluorite 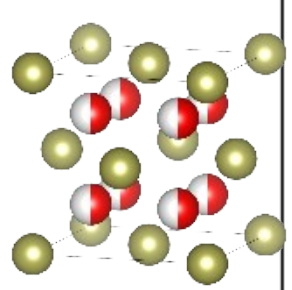	100	37	24.8	3.53
HfO Zincblende (ZnS) 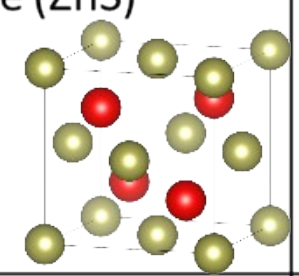	100	72	48.2	1.76
**HfO Rocksalt** 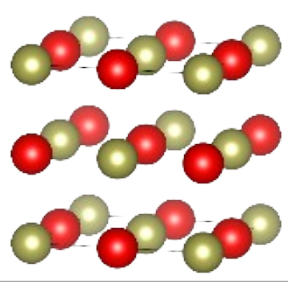	**100**	**68.7**	**30.9**	**1.20**
**Experimental**	**100**	**59**	**35**	**-**
